# Delayed Vision Recovery after Carotid Vascular Surgery for Branch Retinal Artery Occlusion

**DOI:** 10.1155/2019/8243487

**Published:** 2019-01-27

**Authors:** Jessica Ruzicki, Eric K. Chin, David Almeida

**Affiliations:** ^1^Department of Ophthalmology, Kingston Health Sciences Centre, Queen's University, Kingston, ON, Canada; ^2^Private Practice, Retina Consultants of Southern California, Redlands, California, USA; ^3^Private Practice, Metrolina Eye Associates, Charlotte, North Carolina, USA

## Abstract

Branch retinal artery occlusion (BRAO) is typically associated with irreversible vision and peripheral visual field loss. We report a case of a 62-year-old woman with a BRAO related to several cardiovascular risk factors. Our patient encountered gradual but significant vision recovery months following carotid artery endarterectomy for carotid stenosis.

## 1. Introduction

Branch retinal artery occlusion (BRAO) accounts for 38% of acute retinal artery obstruction cases [1]. In BRAO, obstruction of blood flow in the distribution of a branch of the central retinal artery leads to ischemia and reorganization of the retinal layers [2]. Concurrent or sequential cardiovascular events such as stroke or acute myocardial infarct can occur, and thus urgent systemic cardiovascular workup is often warranted [3].

Embolism is the most common cause of retinal artery occlusions with calcific plaques originating from the carotid artery as the primary source and less commonly the aortic arch or heart [3]. Patients with embolic BRAO have a higher frequency of acute cerebral infarctions along with a higher frequency of stenotic carotid arteries compared to arterial occlusions secondary to a nonembolic source [4]. Typical risk factors for embolic arterial occlusions include smoking, concurrent acute ischemic stroke, and carotid artery stenosis.

Various treatment modalities for BRAO have been proposed and reported including YAG laser embolysis and embolectomy, surgical embolus removal, and local fibrinolysis. Unfortunately, none of these have achieved an outcome better than natural history and each can be associated with serious complications [5].

Vision recovery after embolic BRAO is rare. Previously, we reported a case of surgical retinal embolectomy where retinal embolectomy successfully prevented vision loss [6]. Currently, there is little evidence of vision recovery after ocular or extraocular surgical intervention is done. Herein, we report a unique case of a patient with a BRAO who had dramatic but gradual visual improvement following carotid endarterectomy.

## 2. Case Presentation

**Figure 1:**
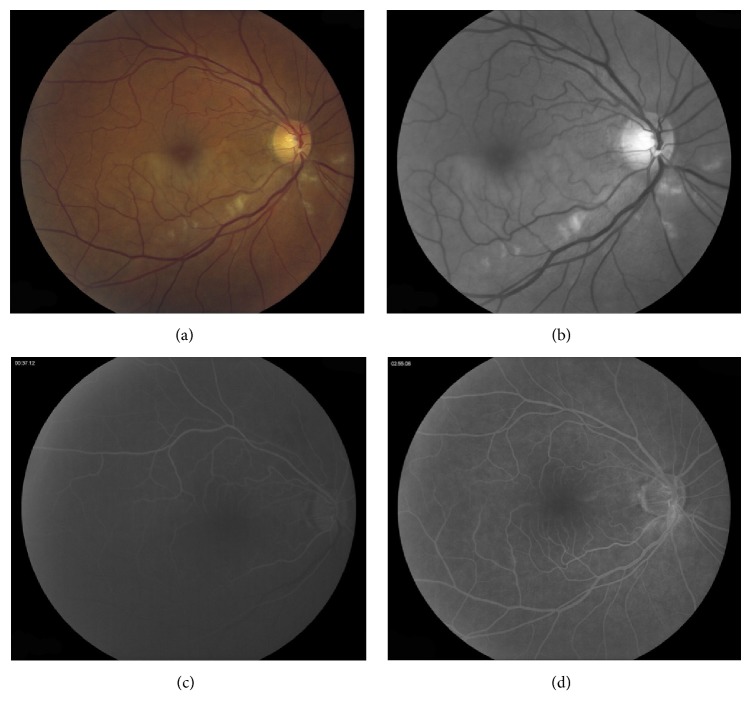
Color fundus photograph of the right eye at initial presentation where BCVA was 20/20 (a) demonstrating the site of occlusion with the emboli situated in a branch of the central retinal artery on the optic disc. Inferior retinal whitening was visible. Red free (b) photograph of the right eye at initial presentation. Fluorescein angiography at 37 seconds (c) showing delayed arterial filling along the inferior retinal vascular arcade. Fluorescein angiography at 2 minutes and 55 seconds (d) showing mild hyperfluroescence along the vessels emanating from the inferior optic nerve.

A 62-year-old woman presented with a one-month history of sudden painless visual loss in the right eye. On examination, best corrected visual acuity (BCVA) was 20/20 in both eyes. Intraocular pressure was 21 mmHg in both eyes. Dilated funduscopic examination in the right eye revealed retinal emboli inferior to the optic disc obstructing a small arteriole associated with retinal ischemia (Figures 1(a) and 1(b)). The left eye was unremarkable. There was no prior past ocular history. The patient had hypertension and hypercholesterolemia and had an extensive smoking history.

**Figure 2:**
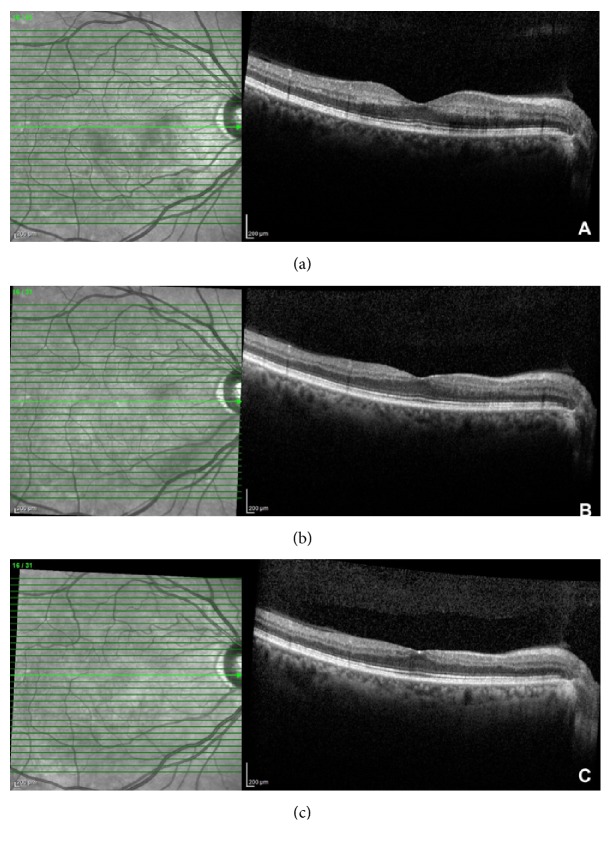
Optical coherence tomography showing acute inner retinal thickening and hyperreflectivity at the patient's initial presentation (a). Optical coherence tomography showing very mild thinning and atrophy of the inner retinal layers two and a half months (b) and six months after her initial presentation (c).

Fluorescein angiography revealed delayed retinal perfusion along the inferior arcade in the right eye (Figures 1(c) and 1(d)). Optical coherence tomography showed normal foveal contour with inner retinal ischemia and thickening, consistent with an acute inferotemporal branch retinal artery occlusion (Figure 2(a)). The patient was started on Latanoprost at nighttime in the right eye to lower the intraocular pressure in hopes to increase reperfusion of the retina. An extensive cardiovascular workup was done, and significant carotid artery stenosis of less than 70% was found. She was started on plavix and aspirin by her cardiologist. The patient continued with BCVA 20/20 OU vision and persistent highly refractile peripapillary emboli; retinal ischemia resolved. Her OCT showed inner retinal atrophy (Figures 2(b) and 2(c)).

**Figure 3:**
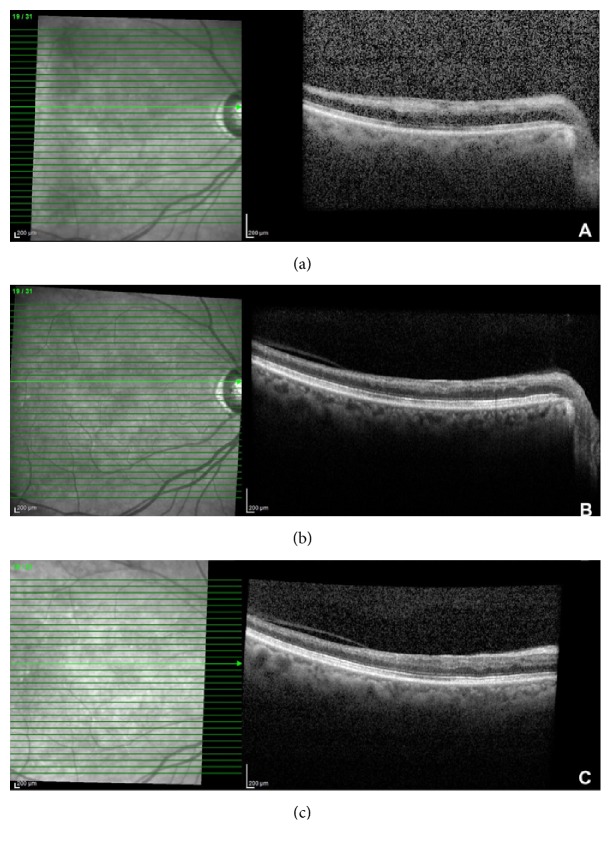
Optical coherence tomography showing inner retinal thickening and hyperreflectivity at the time of acute vision loss, a year and a half following her initial presentation where BCVA was CF (a). Optical coherence tomography imaging showing thinning and atrophy of the inner retina at six months (b) and one year after her acute vision loss secondary to BRAO (c).

Unexpectedly, the patient presented with a three-day history of sudden painless visual loss OD a year and a half after her initial presentation. BCVA was counting fingers (CF) in the right eye. Fundus exam revealed new superotemporal retinal ischemia associated with two new emboli. OCT demonstrated thickening and hyperreflectivity of the inner retinal layers consistent with an acute BRAO OD (Figure 3(a)). The patient refused fluorescein angiography at that time. Urgent workup revealed worsening stenosis to 80% in her right common carotid artery. The vascular surgery referral prompted a right carotid endarterectomy with a carotid stent placement approximately two months following her presentation with acute vision loss.

After endarterectomy, vision in the right eye improved from counting fingers to 20/200 and 20/250 at 2 months and 6 months, respectively. Postoperatively, retinal whitening resolved and reduced intraretinal edema was noticed (Figure 3(b)). One year after endarterectomy, visual acuity was 20/30. The retinal swelling had resolved (Figure 3(c)).

## 3. Discussion

Our patient presented with a clinical picture consistent an embolic BRAO. However, there was significant and gradual improvement in her vision following carotid endarterectomy. Currently, there exists no evidence-based efficacious treatment for BRAO [3]. Our patient demonstrated a varying clinical course with loss of her central vision initially but her vision significantly improved following right carotid endarterectomy surgery.

Carotid endarterectomy has been studied for carotid stenosis; according to the most current Cochrane Review [7], participants defined as symptomatic had stenosis of the ipsilateral (symptomatic) internal carotid artery with recent TIA, nondisabling ischemic stroke, or retinal infarction. Current recommendations indicate that endarterectomy is beneficial for those symptomatic patients with 70% to 99% stenosis and of some benefit to those with 50 to 69% of stenosis (moderate-quality evidence). Further emboli can possibly be released during or after a carotid endarterectomy; therefore care must be taken to remove all of the debris from the intimal surface of the artery.

Dr. Hayreh and Dr. Zimmerman [8] have described an entity called “transient” central retinal arterial occlusion (CRAO) that is based on a sudden vision loss and classic fundus findings of CRAO but normal retinal circulation based on initial fluorescein angiography. An OCT angiography can be performed in unclear cases as well [3]; however this was not available at our institution. Dr. Hayreh and Dr. Zimmerman [8] concluded that CRAO spontaneous improvement in vision and visual field mainly occurs in the first 7 days if any visual recovery is to be seen. However, visual recovery is rare with arterial occlusions.

Experimental studies have shown that the retina suffers no damage with RAOs lasting less than 97 minutes; however, occlusions lasting longer than about 240 minutes cause irreversible retinal damage [9]. With CRAO, Hayreh SS et al. concluded that unless retinal circulation has returned to normal within 4 hours, treatment instituted more than four hours or even days after the onset of CRAO cannot typically achieve any improvement of vision [8].

Our case is unique in that our patient with cardiovascular risk factors had a BRAO with significant acute vision loss. Her endarterectomy was several months after her presentation with acute visual loss, and her vision improvement was gradually seen months after this extraocular intervention. Our case highlights the utility of urgent cardiovascular workup and referral to a subspecialist pending further workup. Long-term vascular remodeling, especially prominent in former smokers, may lead to cardiovascular recovery as a protective recovery mechanism. The exact timing and threshold for endarterectomy in the setting of acute BRAO warrants further investigation.
